# Editorial

**Published:** 1982

**Authors:** 


### Editorial

Dr. Donald Ractliffe, our President in 1979, chose as the title of his Presidential Address 'The Evolution of Medical Societies in Britain – have they a future?' He concluded his address by saying "My own feeling is that, as medical societies, we should further reduce the number of meetings devoted to purely scientific subjects since these tend to duplicate the function of the postgraduate centres. Instead we should establish a forum where we can broaden our learning and thereby compensate for its restrictiveness in our early years." As a profession we are not, by and large, an uncultured lot, but such culture as we have, such learning in the wider sense has been acquired in spite of, rather than because of our medical education. Since 1979 our society has evolved along the lines prescribed by Dr. Ractliffe and the contents of this number are a reflection of this trend; articles on the Bristol Zoo, on Fungi and on Literature – but all with medical connections, and all very good reading. *Floreat Postgraduate Education.*

Another innovation is the column *From our Correspondents.* Seven correspondents have been recruited. They have undertaken to produce a paragraph for each quarterly number on anything that interests them and seems worth saying, they are not to be mere reporters. They represent many of the main branches of our profession, namely, in alphabetical order – General Practice (Dr. Michael Whitfield), Infectious Diseases (Dr. Dennis Wright), General Medicine (Dr. Gordon Mather), Obstetrics and Gynaecology (Professor Gordon Stirrat), Pathology (Dr. Colin Tribe), Radiology (Dr. Gordon Thomson) and Surgery (Professor Robin Williamson). They also represent the University, The Royal Infirmary, The Maternity Hospital, Southmead Hospital, Frenchay Hospital and Ham Green Hospital. While a total coverage of all news is neither possible nor desirable, we may hope that they will tell us most of the things we ought to know, fly kites that ought to be flown and even give their prejudices an airing.

